# Community-based dental education in Iranian dental schools

**DOI:** 10.1186/s12903-024-04290-x

**Published:** 2024-05-03

**Authors:** Ata Bandehagh, Mohammad Reza Khami, Fatemeh Farshad, Hossein Hessari

**Affiliations:** 1 Research Center for Caries Prevention (RCCP), Dentistry Research Institute, Tehran University of Medical Sciences, First floor, Qods Street, Enghelab Avenue, Tehran, 1417614411 Iran; 2 Department of Community Oral Health, School of Dentistry, Tehran University of Medical Sciences, Tehran, Iran

**Keywords:** Community-based dental education, Dental students, Dental education

## Abstract

**Background:**

Community-based dental education (CBDE) has been an essential advancement in dental education in recent decades, enhancing it in many aspects. This study aimed to determine the characteristics and improvements of CBDE in dental schools in Iran.

**Methods:**

In the present descriptive study, an electronic questionnaire, including 18 “yes/no”, “multiple choice”, and “short answer” questions about the nature and extent of CBDE and students’ experience in CBDE, was used. In early 2021, the questionnaires were mailed to the deans of all 43 dental schools in Iran under the supervision of the Council for Dental Education of the Iranian Ministry of Health and Medical Education. Reminder calls were made after 6 and 12 weeks. Dental schools that did not follow the CBDE program were excluded. The responses were analyzed descriptively.

**Results:**

Thirty-six dental schools completed the questionnaire (response rate: 84%). Seventeen schools (47%) reported having CBDE in their dental program. Sites lacking a well-equipped dental setting were the most used out of all extramural sites. The number of weeks dedicated to CBDE ranged between 1 and 20 (median: 4). The most common dental procedures practiced in extramural sites were pediatric dentistry (100%), restorative dentistry (71%), and dental examination (59%). The student-to-supervisor ratio in CBDE ranged between 3 and 15 (median: 5). In most schools (65%), the staff involved in directing CBDE were Community Oral Health PhDs.

**Conclusions:**

An increasing number of Iranian dental schools have integrated CBDE into their undergraduate dental curriculum. The characteristics and extent of this educational strategy vary widely among dental schools. CBDE can be more effective by making positive changes in dental programs.

## Background

Over the past decades, many dentists have graduated from dental schools in Iran. However, the increase in dentists does not sufficiently address disparities in oral care, especially in underserved areas [1]. Prevention and population wellness should be an inner desire in new graduates [2]. Socially conscious dentists are the outcome of an educational system focusing on community issues [3, 4]. As a result, there should be undergraduate dental programs to train dental students to be socially aware.

The Council for Dental Education of the Iranian Ministry of Health and Medical Education in Iran developed a national undergraduate dental curriculum that all dental schools must follow. However, due to the facilities and properties available in schools, there may be minor differences between dental schools’ educational programs.

The undergraduate dental program in Iran takes at least six years, resulting in a DDS degree, which has the same credit allocations across all schools [5]. In the first two years, the students should complete 24 credits for general courses and 39 credits for basic sciences. In the next four years, the students take preclinical and clinical core courses and complete 150 credits for special sciences. Additionally, there are some noncore courses, of which the students should complete 4 out of 30 credits at their discretion.

Community-Based Dental Education (CBDE) and Competency-Based Education have been two critical advances in clinical education in the past 60 years [6]. CBDE program lets dental students complete required clinical competencies based on their clinical experiences [7]. Today, CBDE is a new vision of dental education in dental schools in developed countries [4, 8, 9]. At CBDE rotations, students experience the interaction with and treatment of diverse populations with differences in socioeconomic status and cultural aspects [10, 11]. Many people, especially underserved people, benefit from these dental services.

Several studies have shown the positive influence of CBDE on clinical education, including students’ self-confidence, productivity, time management, communication skills, treatment planning skills, management skills, empathy, self-awareness, cultural awareness, and professionalism [12–18].

Several studies have reported the impact of CBDE on dental graduates’ practice choice, intention to work for underserved populations, and voluntary work [19–28]. In contrast, some studies failed to find such impacts [29–32].

In the last revision of the national dental curriculum in 2018, special attention was given to extramural programs with at least 24 h (6 days) dedicated to needs assessment, health promotion planning, health education, and dental procedures. Of the 24 h in extramural sites, at least 6 h should be dedicated to dental procedures [33].

In the present study, conducted under the supervision of the Council for Dental Education, we intended to determine the characteristics and extent of CBDE in Iranian dental schools using a questionnaire.

## Methods

The present study is a cross-sectional report in Iran and was approved by the Ethics Committee of the School of Dentistry, TUMS (IR.TUMS.DENTISTRY.REC.1399.177)In early 2021, the Council for Dental Education emailed an invitation letter containing the survey link to the deans of all 43 dental schools in Iran (36 public and 7 private dental schools). Dental schools that did not follow the CBDE program were excluded (19 dental schools). In the letter, the deans were asked to assign an individual in the dental school with knowledge and information of the school’s program to participate in the study by filling out the online questionnaire (appendix). If any of the responses were incomplete or contained ambiguous answers, the respondent was called using the contact information obtained at the end of the questionnaire and asked to edit the responses for a higher reliability. Reminder calls were made after 6 and 12 weeks if the questionnaire was completely unanswered. At the end of the 20th week, the survey link was inactivated.

Informed consent forms included voluntary participation, and permission to publish the report was acquired.

At the start of the questionnaire, the study’s aim and its details were described, and then the respondents were asked to consider CBDE status before the COVID-19 pandemic, and whether the pandemic influenced it. CBDE was defined as a program within the undergraduate dental program where dental students may provide any aspect of dental care outside the dental school. In the next stage, an 18-item questionnaire containing yes/no, multiple choice and short answer questions was developed. The goal was to gather information about the following topics: inclusion of CBDE in the dental schools’ undergraduate program, extramural sites offering CBDE, time dedicated to CBDE, dental procedures performed, student/supervisor ratio, faculties involved in directing CBDE and providing dental office management practice, log books and reflections in CBDE.

The questionnaire did not need content validity, since it was an objective comprehensive checklist with no complication variables. However, five experts in community oral health from the School of Dentistry, Tehran University of Medical Sciences, provided feedback and assessed the questionnaire’s face validity and reliability, which was then piloted. The final questionnaire was uploaded to Google Forms, an online survey platform.

SPSS version 26 (IBM crop, Chicago, IL, USA) was served for descriptive analysis.

## Results

Thirty-six out of 43 dental schools completed the questionnaire (response rate 84%). Seventeen dental schools (47%) reported that CBDE was included in their undergraduate dental program.

When asked about the extramural sites, all schools reported using accessible facilities (sites with limited dental equipment, such as primary schools, nursing homes, and special care facilities).

In addition, 47% (*N* = 8) of the 17 schools used community dental clinics, and 18% (*N* = 3) of the 17 schools possessed school-owned dental clinics. The number of available extramural dental clinics among the schools using community clinics (*N* = 8) was one extramural dental clinic in four schools, two in one school, three in one school, and four in two schools.

Six schools (35%) reported that the extramural sites were located far from the community where students lived, while extramural sites were found in the communities where students lived in other schools (65%).

If asked about the educational year when CBDE was offered, 71% reported in the 6th year, 71% in the 5th year, 18% in the 4th year, and 12% in the 3rd year.

**Fig. 1:**
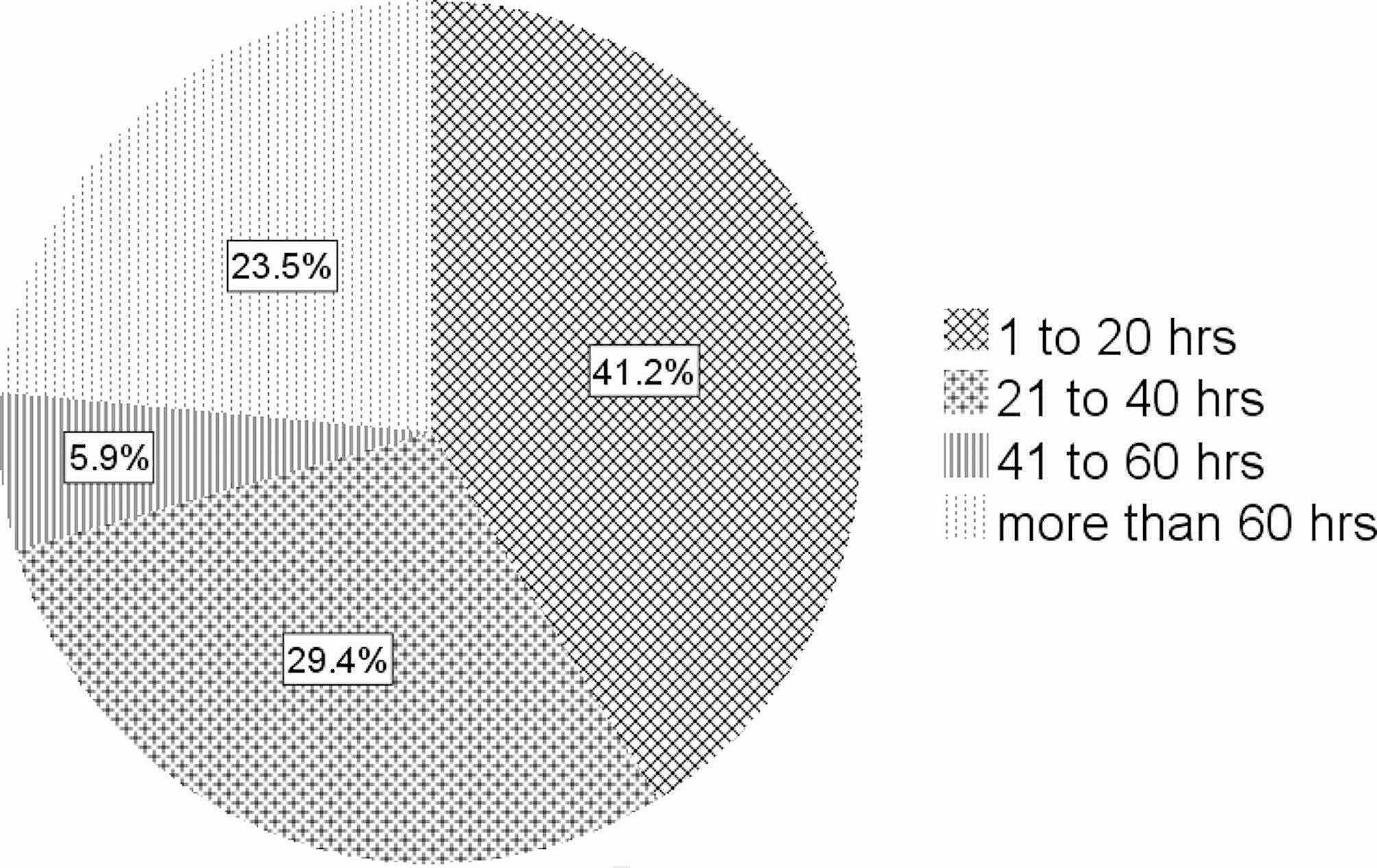
Total hours dedicated to CBDE in dental schools reported by percentage of respondents with extramural programs during a year (*N* = 17) in Iran, 2021

Different lengths of time were dedicated to CBDE. The total hours devoted to CBDE ranged from 6 to 240 h per year (median: 24 h) (Fig. 1). Moreover, the number of weeks dedicated to CBDE ranged between 1 and 20 weeks a year (median: 4 weeks). The number of hours spent on CBDE daily was 3 to 4 h in most schools (88%) and 5 to 6 h in the rest (12%).

**Fig. 2:**
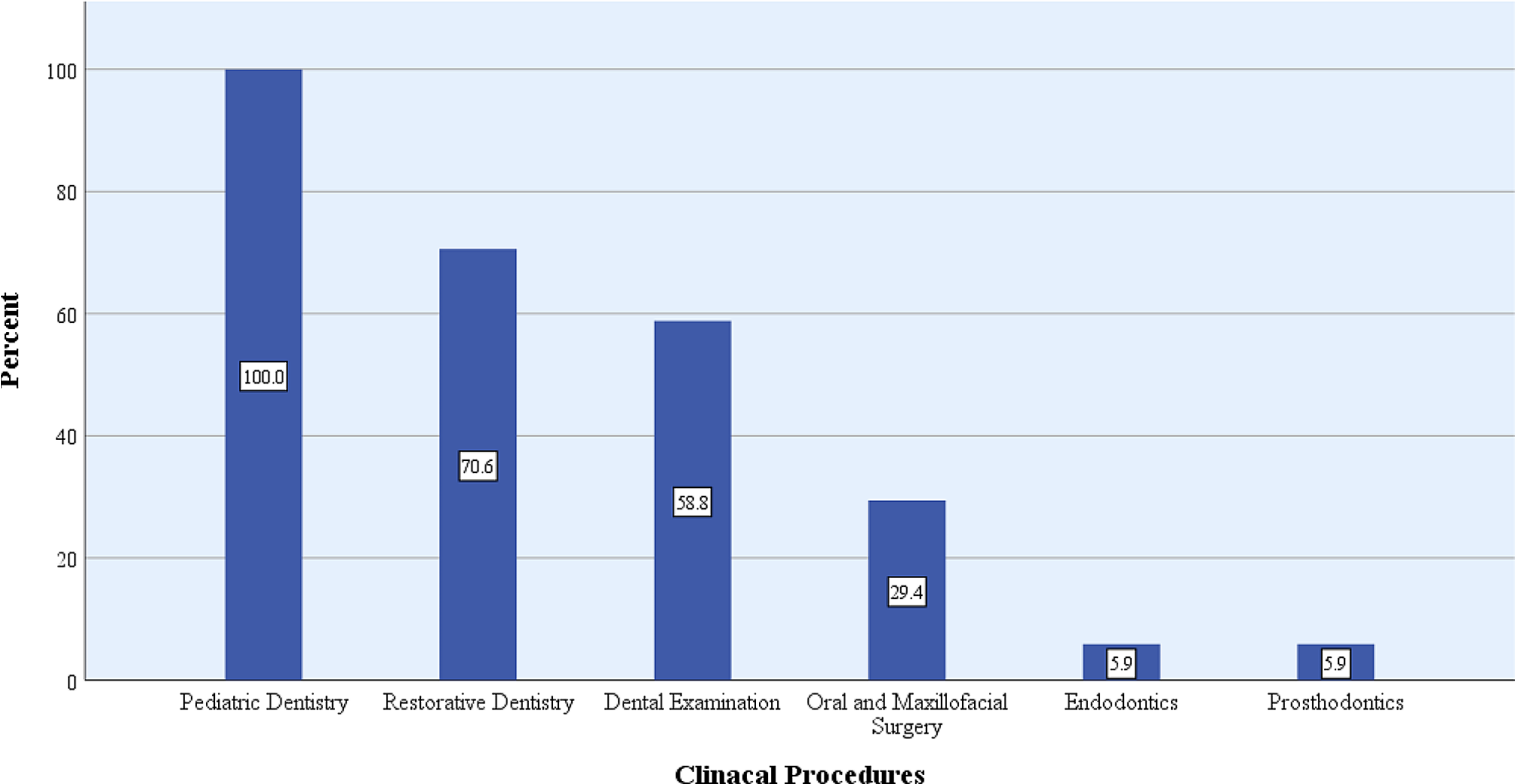
Clinical procedures (%) practiced by dental students at extramural sites, in dental schools having extramural programs (*N* = 17), in Iran, 2021

The most common clinical procedures practiced by dental students in extramural sites were pediatric dentistry (100%), restorative dentistry (71%), and dental examination (59%) (Fig. 2).

**Fig. 3:**
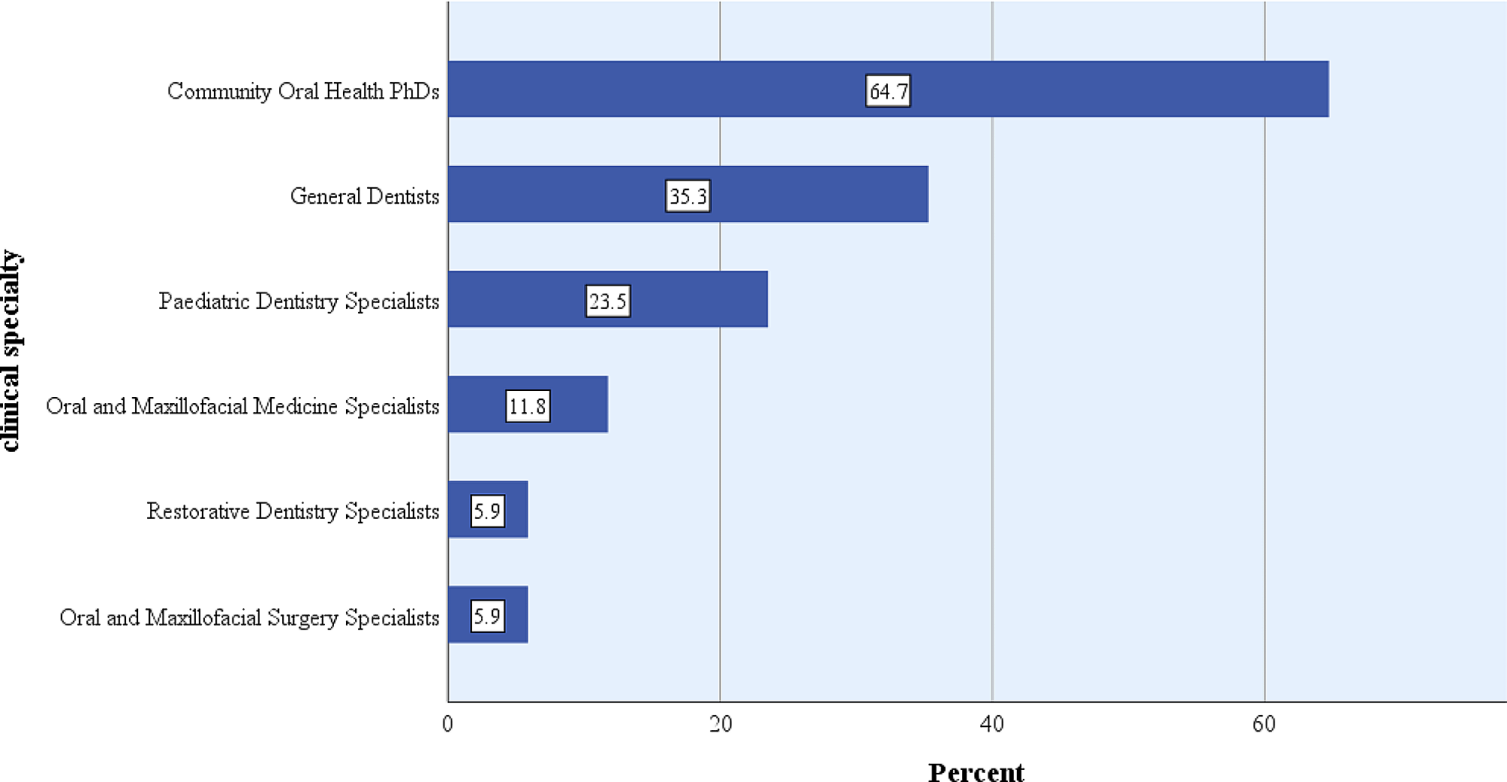
Postgraduate degree of teachers involved in directing CBDE by discipline in dental schools with extramural programs (*N* = 17) in Iran, 2021

The student/supervisor ratio in CBDE ranged between 3 and 15 (median 5). The postgraduate degrees of those involved in directing CBDE varied among schools, including PhDs in community oral health (65%), general dental practitioners (35%), and pediatric dentistry specialists (24%) (Fig. 3).

Five schools (29%) had a course to practice dental office management within the CBDE. Eleven schools (65%) stated that students completed “log books” for procedures performed in CBDE. Fifteen schools (88%) reported that students were asked to mention their opinions about how CBDE is practiced.

## Discussion

The results showed the characteristics of CBDE in Iranian dental schools. Although CBDE is now a part of the undergraduate dental program (since the last revision of the Iranian dental curriculum in 2018), some dental schools still lack this educational method. This may be due to insufficient time for implementing CBDE or other barriers, such as the COVID-19 pandemic. Some of the dental schools have a history of fewer than ten years, indicating many administrative barriers, such as the incomplete implementation of the national curriculum. As a result, some dental schools still need to be improved to meet the minimum requirements for CBDE.

The response rate was high considering the study’s methodology, which was performed in collaboration with the Council for Dental Education. However, attention should be given to the possibility of response bias since schools with organized CBDE were likelier to participate in the present study. In a study in the United States, the response rate was 44% [8]. This difference may result from the national supervision of the Council for Dental Education of the Iran Ministry of Health and Medical Education. The most prevalent sites for CBDE were accessible facilities (the sites lacking a well-equipped dental setting). The accessible facilities varied. Generally, the most selected facilities are primary schools, hospitals, nursing homes, kindergartens, special care facilities, public places, and social counseling centers. There may be several reasons for this finding. First, these sites provide services to more deprived people. Second, many people in Iran receive advanced dental services at dental schools. Third, these sites allow students to serve more individuals at a specific time. Finally, the logistics at these sites are inexpensive for dental schools. The CBDE sites were different in different studies. For instance, in a study in Boston, more than 100 public and private dental sites in twenty-three states were used. Moreover, one or two students were assigned to each site based on the site’s capacity [16]. On the other hand, CBDE rotation sites were categorized into three main clinic types: Hospital, Long-Term Care Facility (LTCF), and others, in the University of Iowa [34].

Dental schools should consider CBDE’s advantages for training students and design their programs to maximize their benefits. Variation in the specified CBDE sites exposes students to diverse patient needs. In sites far from dental schools, the students deal with unprecedented situations and benefit more deprived people, mainly those living in rural areas. CBDE may help students understand the complexity of dental procedures before they graduate.

Preventive-oriented clinical procedures such as pediatric and restorative dentistry were the most prevalent at extramural sites. These procedures (such as fluoride varnish, SDF, and oral health education) are more likely to be performed with limited dental equipment. However, some dental schools may use portable dental units to overcome the limitations of performing more complex dental procedures at such sites. Another prevalent procedure in extramural sites was a dental examination followed by referral of individuals with early and preventable disorders to a dental setting. In addition, in a study conducted at The Ohio State University College of Dentistry, pit and fissure sealants, comprehensive oral examination, posterior one surface resin composite, and topical fluoride treatment were the most prevalent procedures per student [10], which was in line with the present findings.

CBDE is an opportunity for students to experience more diverse dental practices, so it should be implemented in the last years of the undergraduate dental program. In addition, CBDE may significantly impact students’ practice choices in the 5th or 6th year of the undergraduate dental program as they are in more contact with the patients. However, CBDE programs designed longitudinally and run over several years can also benefit students in the early years of undergraduate dental programs [34].

The total time spent at extramural sites was an important variable affecting CBDE. A study reported that dedicating more time to CBDE, especially more than five weeks in different types of extramural sites, may increase the number of students choosing to work in community clinics [35]. Additionally, in another study, dental schools’ net revenue increased with the extended time of CBDE [36]. Furthermore, longer CBDE may increase dental students’ clinical confidence, efficiency, and skills [37].

The number of daily hours spent at extramural sites is another critical aspect of this program. Considering the time needed to commute to external sites, especially rural areas, programs with shorter daily hours will waste more time on the way to these sites. On the other hand, spending a long time at extramural sites may result in student burnout. The present study showed that most dental schools in Iran spent 3 to 4 h daily at extramural sites. However, most dental schools in the U.S., which have a more extended history of CBDE, spent 8 h a day (40 h per week) at extramural sites in 2015 [38].

Dental office management practice, completing logbooks, and producing reflections were ways to enhance dental education via CBDE. CBDE is a valuable opportunity to learn dental office management practice in real situations, especially at clinical sites. Logbooks help students assess themselves regarding CBDE, which may encourage students to be more productive and effective. A well-managed process of reflection enhances the impact of CBDE and promotes the long-term learning of dental students [39].

Dental schools in Iran may need to revise their extramural programs to promote students’ clinical education even more. Further studies should examine the effectiveness of CBDE in dental schools in Iran. Moreover, future studies should focus on difficulties conducting CBDE in dental schools and provide possible solutions.

## Conclusions

CBDE is part of the undergraduate dental curriculum in many dental schools in Iran, but the extent of CBDE varies widely among schools. Schools should consider CBDE to maximize the effectiveness of education.

## Data Availability

The datasets analyzed during the current study are available from the corresponding author on request.

## Appendix

1. Is community-based dental education implemented in your university?

Yes/ No.

If the answer to the first question is “yes”, please answer the following questions.

2. How many hours are devoted to community-based dental education in your university?
3. How is this program implemented?

- Clinics outside the school.
- Clinics built in for this purpose.
- Accessible facilities.
- Others (please specify) ____________.

4. How many types of clinics outside of the university is your university related with?
5. Do students live in the same community where community-based dental education is implemented?

Yes/ No.

6. What is the level of education and expertise of the people managing this program?
7. When do the students participate in community-based dental education?

3th year/ 4th year/ 5th year/ 6th year.

8. What clinical procedures are delivered in this program?

- Pediatric dentistry.
- Prosthodontics.
- Restorative dentistry.
- Endodontics.
- Orthodontics.
- Oral and maxillofacial surgery.
- Dental examination.

9. Who participates in community-based dental education?

- All students.
- Top students.
- Volunteer students.
- Others (please specify) __.

10. How many weeks do the students practice in the extramural sites in total? ____weeks.
11. How many days per week do students practice in the extramural sites? ____ days.
12. How many hours daily do the students practice in the extramural sites? ____hours__________.
13. What is the ratio of students to supervisors in your program?
14. Is dental office management practiced in extramural sites?

Yes/No.

15. Are the students evaluated in community-based dental education?

Yes/No.

16. Do the students fill out the log book?

Yes/No.

17. Are there any feedback at the end of rotations?

Yes/No.

18. What are the current challenges for your community-based dental education programs?

## Abbreviations

COH: Community Oral Health
CBDE: Community-Based Dental Education
